# Effect of Difference in Consensus Sequence between HIV-1 Subtype A/E and Subtype B Viruses on Elicitation of Gag-Specific CD8^+^ T Cells and Accumulation of HLA-Associated Escape Mutations

**DOI:** 10.1128/JVI.02061-20

**Published:** 2021-02-24

**Authors:** Yu Zhang, Hayato Murakoshi, Takayuki Chikata, Tomohiro Akahoshi, Giang Van Tran, Trung Vu Nguyen, Hiroyuki Gatanaga, Kinh Van Nguyen, Shinichi Oka, Nozomi Kuse, Masafumi Takiguchi

**Affiliations:** a Tokyo Joint Laboratory and Division of International Collaboration Research, Joint Research Center for Human Retrovirus Infection, Kumamoto University, Kumamoto, Tokyo, Japan; b Center for AIDS Research, Kumamoto University, Kumamoto, Japan; c National Hospital of Tropical Diseases, Hanoi, Vietnam; d Hanoi Medical University, Hanoi, Vietnam; e AIDS Clinical Center, National Center for Global Health and Medicine, Tokyo, Japan; Emory University

**Keywords:** CTL, escape mutation, HIV-1, subtype A/E, subtype B

## Abstract

The Gag280 mutation is associated with HLA-C*01:02 but not with HLA-B*52:01 in subtype A/E-infected individuals, whereas this mutation is associated with HLA-B*52:01 but not with HLA-C*01:02 in subtype B infections. Although it is known that the Gag280 mutant is selected by HLA-B*52:01-restricted GagRI8 (Gag275-282)-specific T cells in subtype B infections, it remains unknown why this Gag280 mutation is associated with HLA-C*01:02 rather than HLA-B*52:01 in subtype A/E infections. The subtype B and A/E viruses have different consensus sequence, with Thr and Val at Gag280, respectively. To clarify the effect of this difference in Gag280 consensus sequence, we investigated the role of HLA-C*01:02-restricted GagYI9 (Gag277-285)-specific T cells in selection of Gag280 mutations in subtype A/E-infected Vietnamese and subtype B-infected Japanese individuals. GagYI9-4V-specific T cells, which were frequently elicited in Vietnamese individuals infected with the consensus-type A/E virus, failed to recognize GagV280T mutant A/E virus-infected cells. GagYI9-4T mutant epitope-specific T cells, which were weakly elicited in individuals infected with the mutant A/E virus, had weak or no ability to recognize the mutant virus. These results account for the mechanism for selection and accumulation of GagV280T mutants in the case of subtype A/E infections. In contrast, HLA-C*01:02-restricted GagYI9-4T-specific T cells were weakly elicited in Japanese individuals infected with the subtype B virus, explaining why HLA-C*01:02-restricted Gag280 mutations are not accumulated in the case of a subtype B infection. The present study demonstrated that a difference in the Gag280 consensus sequence influenced the elicitation of the GagYI9-specific T cells involved in the accumulation of HLA-C*01:02-associated Gag280 mutations.

**IMPORTANCE** HIV-1 mutations escaped from HIV-specific CD8^+^ T cells are mostly detected as HLA-associated mutations. A diversity of HLA-associated mutations is somewhat distinct to each race and region, since HLA allele distribution differs among them. A difference in the consensus sequence among HIV-1 subtypes may also influence the diversity of HLA-associated mutations. HLA-C*01:02-associated GagV280T and HLA-B*52:01-associated GagT280A/S mutations were previously identified in HIV-1 subtype A/E-infected and subtype B-infected individuals, respectively, though these subtype viruses have a different consensus sequence at Gag280. We demonstrated that the GagV280T mutant virus was selected by HLA-C*01:02-restricted GagYI9-4V-specific T cells in subtype A/E-infected Vietnamese but that HLA-C*01:02-restricted GagYI9-4T-specific T cells were weakly elicited in subtype B-infected Japanese. Together with our recent study which demonstrated the mechanism for the accumulation of HLA-B*52:01-associated mutations, we clarified the mechanism for the accumulation of different Gag280 mutations and the effect of the difference in the consensus sequence on the accumulation of escape mutations.

## Abstract (precis)

HIV-1 mutations escaped from HIV-specific CD8^+^ T cells are mostly detected as HLA-associated mutations. A diversity of HLA-associated mutations is somewhat distinct to each race and region, since HLA allele distribution differs among them.

## INTRODUCTION

Human leukocyte antigen (HLA) class I-restricted HIV-1-specific CD8^+^ T cells can select viruses having mutations, which affects T-cell recognition in the context of the HLA class I alleles expressed by the host (1–6). HIV sequences circulating in a given population exhibit polymorphisms that reflect the HLA allele distribution in that population (7–9). Numerous population-based studies of HLA-associated polymorphisms (HLA-APs) have been performed in many countries and ethnic groups in Europe, North America, Australia, Asia, and Africa (8, 10–14). Since HLA class I allele distributions differ among racial and ethnic groups worldwide (15), the pattern and diversity of HLA-APs are somewhat distinct to each race and region (9, 13). In addition, a difference in sequences among HIV-1 subtypes may affect the diversity of HLA-APs (16). Thus, information about HLA-APs among different HIV-1 subtypes will be useful for development of a universal HIV-1 vaccine.

HLA-B27/B57 and HLA-B*52:01 are well known to be protective alleles in HIV-1 infections (17–21). HLA-B27/B57-associated mutations are found in protective epitopes restricted by HLA-B27/B57 alleles among Caucasian and African individuals (12, 22–25). These mutations critically affect suppression of HIV-1 replication by T cells specific for these protective epitopes (4, 26–28), suggesting a critical effect of these mutations on HIV-1 control by T cells specific for these protective epitopes. On the other hand, recent studies showed that HLA-APs are not present in 10 of 11 protective T-cell epitopes among HIV-1 subtype B-infected Japanese individuals (13, 29). Thus, the accumulation of escape mutations in the protective epitopes is a rare event in subtype B-infected Japanese individuals. HLA-B*52:01-associated mutations are found at GagT280A/S only within an HLA-B*52:01-restricted GagRI8 (Gag275-282: RMYSPTSI) protective epitope in subtype B-infected individuals (13, 30). A recent study demonstrated that GagRI8 epitope-specific T cells fail to recognize target cells infected with GagT280A/S mutant viruses but that HLA-B*52:01^+^ individuals infected with GagT280A/S mutant viruses do not elicit T cells specific for these mutant epitopes (31), suggesting that these escape mutants are selected by the GagRI8-specific T cells in subtype B-infected Japanese individuals.

**FIG 1:**
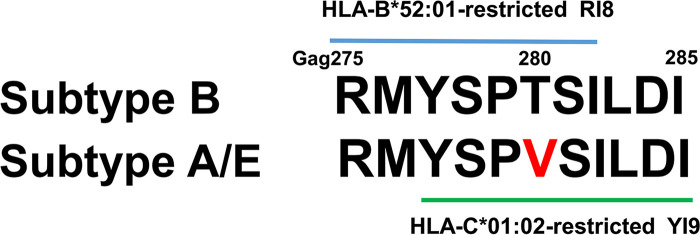
Gag275-285 consensus sequence in HIV-1 subtype B and A/E virus. HLA-C*01:02-restricted YI9 and HLA-B*52:01-restricted RI8 epitopes are included in this sequence.

Although the GagT280V mutation is also found in approximately 10% of subtype B-infected HLA-B*52:01^+^ Japanese individuals, it is not an HLA-B*52:01-associated mutation (13). A recent study showed that HLA-B*52:01^+^ Japanese individuals infected with the GagT280V mutant virus effectively elicit RI8-6V mutant-specific T cells and that GagT280V mutant and Gag280 consensus-type (GagT280) subtype B viruses are selected by GagRI8-specific and GagRI8-6V mutant-specific T cells, respectively, indicating a mechanism by which GagT280V mutant viruses are not accumulated in HLA-B*52:01^+^ Japanese individuals (31). A previous study demonstrated that the HLA-C*01:02-associated GagV280T mutation is detected in Vietnamese individuals infected with the subtype A/E virus carrying a consensus sequence with Val at Gag280 (GagV280); however, an HLA-B*52:01-associated mutation at Gag280 could not be analyzed in these individuals due to the low frequency of HLA-B*52:01 (32). These findings imply that this HLA-C*01:02-associated GagV280T mutation may be selected by HLA-C*01:02-restricted T cells rather than by HLA-B*52:01-restricted T cells in Vietnamese individuals. A previous study showed that HLA-C*01:02-restricted GagYI9-4V (Gag277-285: YSPVSILDI)-specific T cells, which were established from an HLA-C*01:02^+^ Japanese individual infected with the HIV-1 subtype A/E virus, recognized a GagYI9-4T mutant peptide less effectively than it did a GagYI9-4V peptide, implying that the GagYI9-4V-specific T cells selected for the GagV280T mutant (14). Since the recognition by T cells of target cells infected with the GagV280T mutant virus was not examined in this study (14), it still remains unknown whether the GagV280T mutant is selected by the T cells specific for this epitope in the HLA-C*01:02^+^ Vietnamese. The YI9 and RI8 epitopes are included in the 11-mer peptides (Gag275-285) covering Gag280, where a difference in consensus sequence is found between subtype B and subtype A/E viruses (Fig. 1). HIV-1 subtype A/E and subtype B are dominant in Vietnam and Japan, respectively. This difference between these two subtypes may influence the accumulation of escape mutations at both countries.

In the present study, we investigated the elicitation of HLA-C*01:02-restricted YI9-specific T cells in subtype A/E virus-infected Vietnamese individuals and the selection of the GagV280T mutation by these T cells. We further investigated the elicitation of HLA-C*01:02-restricted YI9-specific T cells in the subtype B virus-infected Japanese individuals and HLA-B*52:01-restricted RI8-specific T cells in the subtype A/E virus-infected Vietnamese. The present study clarified a mechanism for the accumulation of the HLA-C*01:02-associated Gag280T mutation in the subtype A/E virus-infected Vietnamese but not in the subtype B-infected Japanese and further demonstrated the effect of the difference in the consensus sequence at Gag280 between these two subtypes on the accumulation of different escape mutations.

## RESULTS

### Selection of GagV280T mutant virus by YI9-4V-specific CD8^+^ T cells in HLA-C*01:02^+^ Vietnamese individuals infected with HIV-1 subtype A/E.

**FIG 2:**
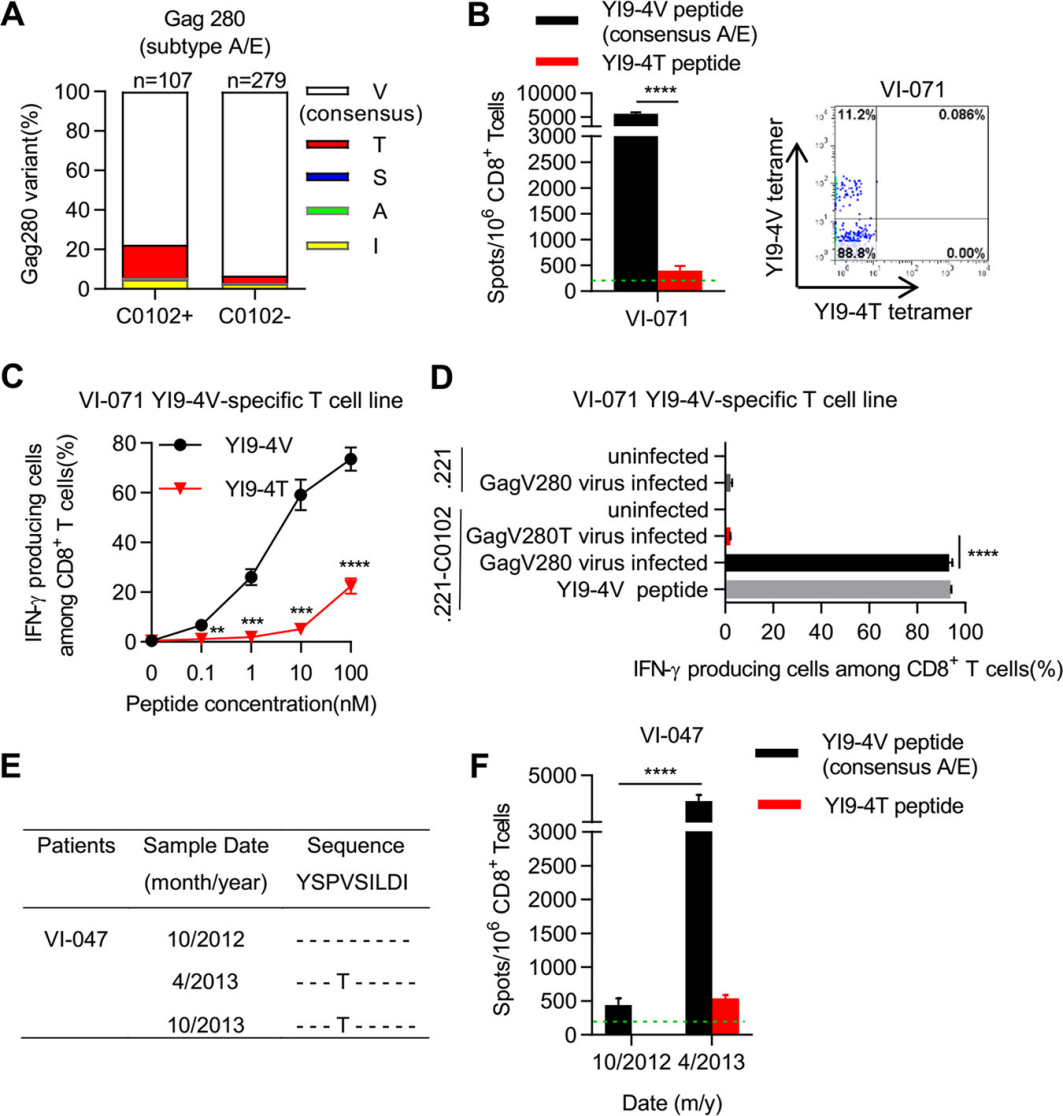
Accumulation and selection of YI9-4T mutation by HLA-C*01:02-restricted YI9-4V-specific T-cells. (A) Mutations at Gag280 in 386 HIV-1 subtype A/E-infected Vietnamese individuals. Amino acid sequence at Gag280 was compared between 107 HLA-C*01:02^+^ individuals and 279 HLA-C*01:02^–^ individuals. T, S, A, I, and V were found at Gag280 in 18, 0, 1, 5, and 83 HLA-C*01:02^+^ individuals, respectively, whereas T, S, A, I, and V were detected at Gag280 in 11, 0, 1, 7, and 260 HLA-C*01:02^–^ individuals, respectively. (B) Response and identification of YI9-specific T cells in PBMCs from an individual (VI-071) infected with the GagV280 virus. The T cell response to YI9-4V or YI9-4T peptide was measured by using an ELISPOT assay (left). YI9 epitope-specific T cells in CD3^+^CD8^+^ cells were identified by staining PBMCs from patient VI-071 with HLA-C*01:02-YI9-4V and HLA-C*01:02-YI9-4T tetramers (right). The percentages of tetramer-positive cells among CD3^+^ CD8^+^ 7-AAD^–^ T cells were measured (right). (C and D) Recognition of YI9-4T mutant epitope by a YI9-4V-specific T-cell line. The T-cell response to 721.221-C*01:02 cells prepulsed with YI9-4V or -4T peptide (C) and to those infected with 93JP-NH1-GagV280 or -GagV280T were analyzed by performing an intracellular cytokine staining (ICS) assay (D). The frequencies of p24 antigen-positive cells among 721.221-C*01:02 cells infected with 93JP-NH1-GagV280 and -GagV280T were 26.6 and 33.9%, respectively, whereas the frequency of 721.221-C*01:02 cells infected with 93JP-NH1-GagV280 was 21%. (E) Longitudinal sequence analysis at Gag280 in an HLA-C*01:02^+^ B*52:01^–^ Vietnamese individual (VI-047) infected with the HIV-1 subtype A/E virus. (F) Longitudinal analysis of T-cell responses to YI9-4V peptide or -4T mutant peptide in individual VI-047. The dotted line at 200 spots/10^6^ CD8^+^ T cells indicates a threshold for a positive response (B and F). All data are presented as means and SD (*n* = 3). Statistical analysis was performed with the unpaired *t* test (B, C, D, and F), and the results are indicated by asterisks (**, *P* < 0.01; ***, *P* < 0.001; ****, *P* < 0.0001).

A previous study showed that the HLA-C*01:02-associated mutation GagV280T accumulates in subtype A/E-infected Vietnamese individuals (14). To confirm this result, we identified Gag sequences in 21 Vietnamese individuals chronically infected with the subtype A/E and then reanalyzed HLA-C*01:02-associated mutation by using Gag 280 sequence data from a total of 386 HIV-1 subtype A/E-infected Vietnamese, which data included that from a previous analysis (14). The results showed that GagV280T accumulated in the subtype A/E-infected HLA-C*01:02^+^ Vietnamese individuals (*P = *5.91 × 10^−5^, *q* = 8.86 × 10^−5^) but that GagV280A and GagV280S were very rarely detected in them (Fig. 2A).

We previously revealed that T cells specific for the HLA-C*01:02-restricted YI9-4V epitope, which cells were established from 2 HLA-C*01:02^+^ Japanese individuals infected the subtype A/E virus, recognized the YI9-4V peptide much more effectively than the YI9-4T one (14). However, it remains unknown whether YI9-4V-specific T cells are elicited in subtype A/E-infected HLA-C*01:02^+^ Vietnamese individuals. We therefore sought to identify HLA-C*01:02-restricted YI9-4V-specific T cells in the subtype A/E-infected HLA-C*01:02^+^ Vietnamese individuals. We investigated the existence of HLA-C*01:02-restricted YI9-specific T cells in an HLA-C*01:02^+^B*52:01^−^ Vietnamese individual infected with GagV280 consensus-type subtype A/E virus (VI-071) by performing enzyme-linked immunosorbent spot (ELISPOT) analysis and flow cytometry analysis using both HLA-C*01:02-YI9-4V and HLA-C*01:02-YI9-4T tetramers. The results of the ELISPOT and flow cytometry analyses showed a strong T-cell response to the YI9-4V peptide but not to the YI9-4T one (Fig. 2B, left) and the existence of a high number of HLA-C*01:02-restricted YI9-4V-specific T cells (Fig. 2B, right), respectively. Since our previous study did not show recognition by YI9-4V-specific T cells of target cells infected with the GagV280T mutant virus, we next investigated whether the YI9-4V-specific T cells could recognize target cells infected with the GagV280T mutant virus. We established a YI9-4V-specific T-cell line from patient VI-071 and analyzed the recognition by this T-cell line of target cells prepulsed with YI9-4V or YI9-4T peptide and those infected with the consensus-type subtype A/E virus (93JP-NH1) or Gag V280T mutant one (93JP-NH1-GagV280T). The T-cell line strongly recognized 721.221 cells expressing HLA-C*01:02 (721.221-C*01:02 cells) prepulsed with the YI9-4V peptide and very weakly those prepulsed with the YI9-4T mutant one (Fig. 2C), whereas they recognized 721.221-C*01:02 cells infected with the consensus-type virus but not those infected with the mutant one (Fig. 2D). These findings taken together indicate that GagV280T was a mutation that had escaped from HLA-C*01:02-restricted YI9-4V-specific T cells.

We next analyzed an HLA-C*01:02^+^ B*52:01^−^ Vietnamese individual, VI-047, who exhibited the V to T substitution at Gag280 for over 6 months. The consensus-type subtype A/E virus was found in October 2012, and then the GagV280T mutant was detected in April 2013 and October 2013 in this individual (Fig. 2E). We performed a longitudinal analysis of YI9-4T/V-specific T cells. A T-cell response to the YI9-4V peptide was detected in October 2012, and an increase in the response to this peptide was found in April 2013, whereas the T-cell response to the YI9-4T mutant peptide was weakly detected in April 2013 at the emergence of the GagV280T mutant virus (Fig. 2F). These findings suggest that the GagV280T mutation could be selected by YI9-4V-specific T cells in this individual.

### Recognition of consensus-type and GagV280T mutant viruses by YI9-4T-specific, YI9-4V-specific, and cross-reactive T cells.

**FIG 3:**
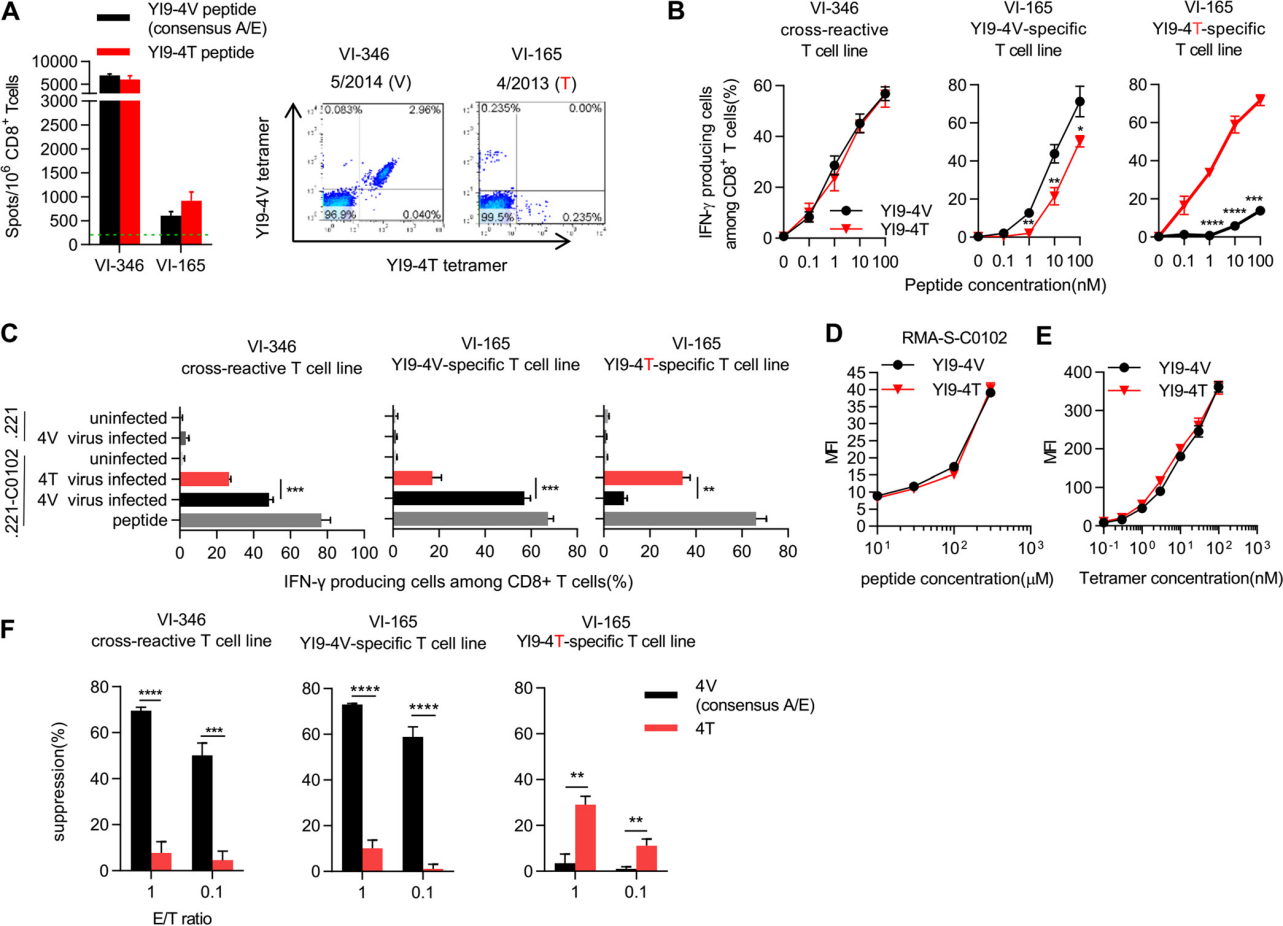
Recognition of YI9-4T mutant epitope by HLA-C*01:02-restricted cross-reactive and YI9-4T-specific T cells. (A) Response and identification of YI9 epitope-specific T cells among PBMCs from VI-346 and VI-165 individuals infected with the consensus-type and GagV280T mutant virus, respectively. The T cell response to YI9-4V or YI9-4T peptide was measured by using the ELISPOT assay (left). YI9 epitope-specific T cells were identified by flow cytometry analysis using HLA-C*01:02-YI9-4V and HLA-C*01:02-YI9-4T tetramers (right). The dotted line at 200 spots/10^6^ CD8^+^ T cells indicates the threshold for a positive response (left). (B and C) Recognition of YI9-4V or -4T epitope by YI9-4V-specific, YI9-4T-specific, or cross-reactive T cells. The cross-reactive T-cell line was established from individual VI-346, whereas YI9-4V-specific and -4T-specific ones were established from individual VI-165. Responses of these T-cell lines to 721.221-C*01:02 cells prepulsed with YI9-4V or YI9-4T peptide (B) and to those infected with 93JP-NH1-GagV280 or -GagV280T (C) were analyzed by using the ICS assay. YI9-4V and YI9-4T peptides were used as a control for YI9-specific and cross-reactive T cells and for YI9-4T-specific T cells, respectively (C). The frequencies of p24 antigen-positive cells among 721.221-C*01:02 cells infected with 93JP-NH1-GagV280 or -GagV280T were 20 or 20.8%, respectively, and those of 721.221-C*01:02 cells infected 93JP-NH1-GagV280 or -GagV280T were 16.3 and 18.6%, respectively. (D) Binding of YI9-4V and YI9-4T epitope peptides to HLA-C*01:02. Binding affinity was measured by use of the HLA class I stabilization assay using RMA-S-C*01:02 cells. (E) Binding ability of the cross-reactive T-cell line to HLA-C*01:02-YI9-4V and HLA-C*01:02-YI9-4T tetramers. The cross-reactive T cell line from individual VI-346 was stained with HLA-C*01:02-YI9-4V tetramer or HLA-C*01:02-YI9-4T tetramer at concentrations of 0.1 to 100 nM. (F) Ability of YI9-4V-specific, YI9-4T-specific or cross-reactive T cell lines to suppress the replication of Gag280-4V and Gag280-4T viruses. The percent suppression of HIV-1 replication is presented. All data are shown as means and SD (*n* = 3). Statistical analysis was performed by using the unpaired *t* test (B, C, and F). *, *P* < 0.05; **, *P* < 0.01; ***, *P* < 0.001; ****, *P* < 0.0001.

We next investigated the elicitation of YI9-4T mutant-specific T cells in HLA-C*01:02^+^ Vietnamese individuals infected with the GagV280T mutant virus. We selected two individuals, VI-346 and VI-165, who had been infected with GagV280 consensus-type and GagV280T mutant viruses, respectively. These individuals had positive T-cell responses to both YI9-4V and YI9-4T peptides in the ELISPOT assay (Fig. 3A, left). Flow cytometry analysis using YI9-4V- and YI9-4T-HLA-C*01:02 tetramers showed that VI-165 had both YI9-4V-specific and YI9-4T-specific T cells but that VI-346 had only cross-reactive T cells (Fig. 3A, right). To investigate the ability of these T cells to recognize cells infected with the GagV280T virus, we established cross-reactive T-cell lines from patient VI-346 as well as YI9-4V-specific and YI9-4T-specific T-cell lines from VI-165. Cross-reactive T cells evenly recognized both peptides (Fig. 3B). The YI9-4V-specific T-cell line recognized both YI9-4V and YI9-4T peptides, though this T-cell line recognized the former peptide more effectively than the latter one, whereas the YI9-4T-specific T-cell line recognized the YI9-4T peptide much more so than the YI9-4V one (Fig. 3B).

We next investigated the ability of these T-cell lines to recognize GagV280T virus-infected cells. Cross-reactive and YI9-4V-specific T-cell lines recognized both 721.221-C*01:02 cells infected with the GagV280 virus and those with the GagV280T virus, though they recognized the former cells much more strongly than the latter ones (Fig. 3C, left and middle). In contrast, YI9-4T-specific T cells recognized target cells infected with the GagV280T virus but very weakly those infected with the consensus-type one (Fig. 3C, right). The HLA class I stabilization assay using RMA-S-C*01:02 cells demonstrated that YI9-4V and YI9-4T peptides had very similar binding affinities for HLA-C*01:02 molecules (Fig. 3D). In addition, the tetramer binding assay using HLA-C*01:02-YI9-4V and HLA-C*01:02-YI9-4T tetramers gave results indicating that the cross-reactive T cells had the same TCR affinity for these tetramers (Fig. 3E). These results taken together showed that the cross-reactive T cells had TCRs with the same affinity for HLA-C*01:02-YI9-4V and HLA-C*01:02-YI9-4T. Since these cross-reactive T cells recognized the cells infected with the GagV280 virus more effectively than those infected with the GagV280T one, it is likely that YI9-4T peptide could be less presented in GagV280T virus-infected cells than YI9-4V in GagV280 virus-infected cells. These results together suggest that the GagV280T mutation may have partially affected antigen presentation of this epitope in the cells infected with this mutant virus.

We finally analyzed the ability of these T cells to suppress the replication of the consensus-type virus or GagV280T mutant one *in vitro*. The cross-reactive T cells and YI9-4V-specific T cells effectively suppressed the replication of GagV280 virus but not that of the GagV280T one, whereas YI9-4T-specific T cells weakly suppressed the replication of GagV280T virus but not that of GagV280 one (Fig. 3F). Thus, YI9-4T-specific T cells had a weak ability to suppress the replication of GagV280T mutant virus, suggesting that these T cells could not select for the consensus-type virus.

### Elicitation of YI9-4V/4T-specific T cells in subtype A/E virus-infected HLA-C*01:02^+^ Vietnamese individuals.

**FIG 4:**
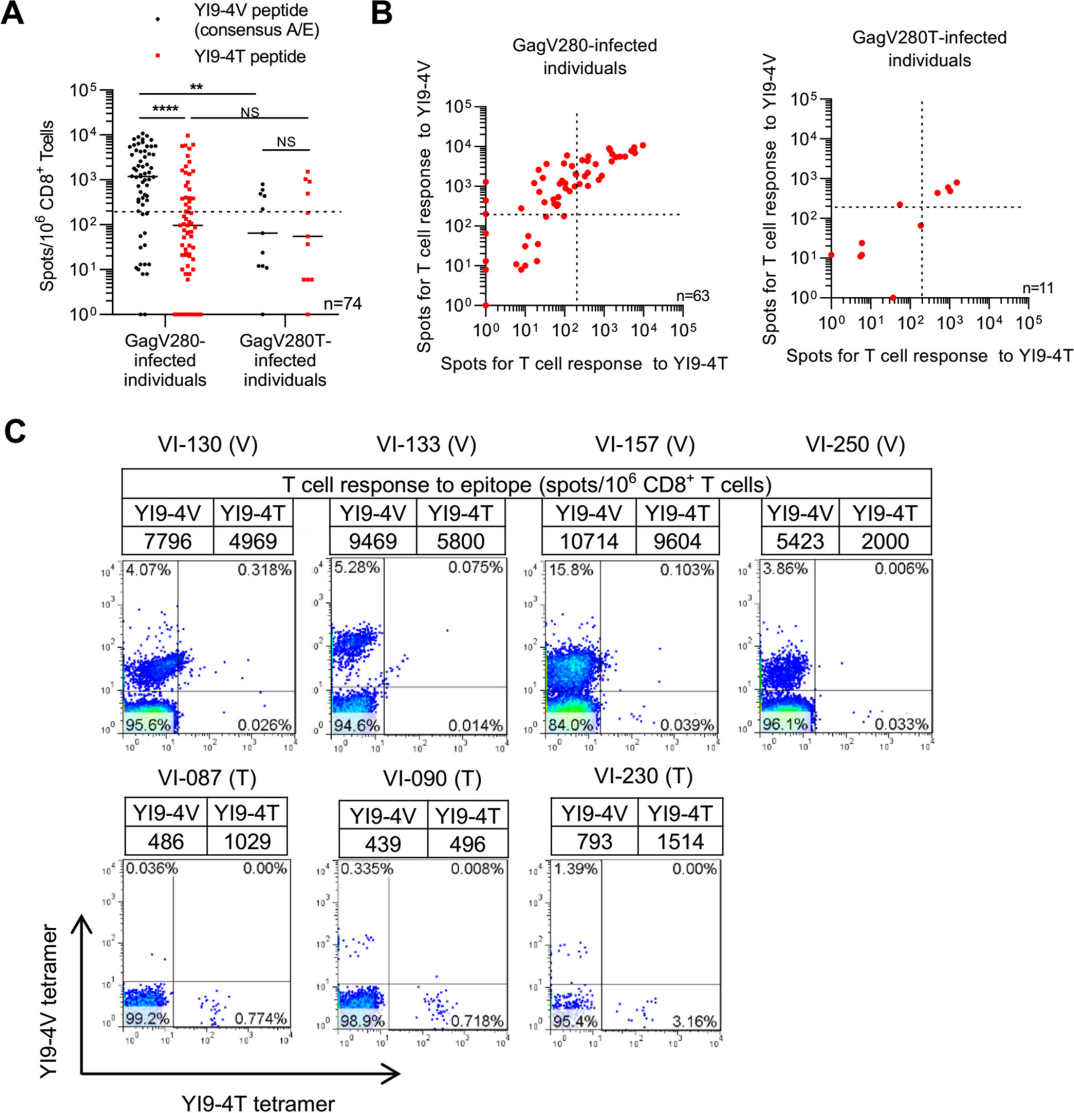
YI9-4V and/or YI9-4T epitope in the subtype A/E virus-infected Vietnamese individuals. (A) T cell responses to YI9-4V peptide or the YI9-4T mutant one in 63 GagV280 virus-infected and 11 GagV280T virus-infected HLA-C*01:02^+^HLA-B*52:01^–^ Vietnamese individuals were analyzed by performing an ELISPOT assay. T-cell responses to the peptide at a concentration of 100 nM were measured. The dotted line at 200 spots/10^6^ CD8^+^ T cells indicates the threshold for a positive response. (B) T-cell responses to the YI9-4V peptide and the YI9-4T mutant in each individual. (C) Identification of YI9 epitope-specific T cells in PBMCs from four GagV280 virus-infected (upper) and three GagV280T virus-infected individuals (lower). PBMCs were stained with HLA-C*01:02-YI9-4V and -YI9-4T tetramers. The T-cell response to YI9-4V and -4T mutant epitope peptides in each individual are shown as the number of spots/10^6^ CD8^+^ T cells. Statistical analysis was performed with the Mann-Whitney test, and the results are indicated (**, *P* < 0.01; ****, *P* < 0.0001; NS, not significant).

We next investigated T-cell responses to the YI9-4V peptide or YI9-4T one in a large number of subtype A/E-infected HLA-C*01:02^+^ B*52:01^–^ Vietnamese individuals. We analyzed 74 Vietnamese individuals (63 consensus-type subtype A/E virus-infected and 11 GagV280T mutant virus-infected HLA-C*01:02^+^ ones) by performing an ELISPOT assay. The numbers (frequency) of responders to YI9-4V and to YI9-4T were 47 (74.6%) and 22 (34.9%), respectively, in the 63 consensus-type virus-infected individuals, whereas those to YI9-4V and YI9-4T were 5 (45.5%) and 4 (36.4%), respectively, in 11 GagV280T virus-infected individuals. The frequency and magnitude of T-cell responses to the YI9-4V peptide in the GagV280 virus-infected individuals were stronger than those to the YI9-4T mutant one in GagV280T virus-infected and GagV280 virus-infected ones (Fig. 4A), indicating that YI9-4T-specific T cells were less effectively elicited in GagV280T virus-infected individuals. These results indicate that the YI9-4T peptide was much less presented to T cells in GagV280T virus-infected individuals. A weak presentation of the YI9-4T epitope peptide by HLA-C*01:02 in GagV280T virus-infected cells would result in weaker elicitation of YI9-4T-specific T cells in the GagV280T virus-infected HLA-C*01:02^+^ individuals.

It should be noted that some HLA-C*01:02^+^ individuals exhibited T cell responses to both YI9-4V and YI9-4T peptides (Fig. 4B). From these findings, we speculated that these individuals had (i) cross-reactive T cells recognizing both peptides, as shown in VI-346, and/or (ii) both YI9-4V-specific and YI9-4T-specific T cells, as shown in the case of patient VI-165. To clarify this, we selected four consensus-type virus-infected and three GagV280T mutant virus-infected individuals who had responded to both peptides and then analyzed peripheral blood mononuclear cells (PBMCs) from these individuals by using HLA-C*01:02-YI9-4V and HLA-C*01:02-YI9-4T tetramers. Both HLA-C*01:02-YI9-4V tetramer-binding and HLA-C*01:02-YI9-4T tetramer-binding T cells were found in all three GagV280T virus-infected individuals (Fig. 4C). The results from these three individuals and VI-165 showed that both YI9-4T-specific and YI9-4V-specific T cells were elicited in the GagV280T virus-infected responders who showed T-cell responses to both peptides by the ELISPOT assay. The YI9-4V-specific T cells in these individuals may have been memory T cells elicited during a phase of the consensus-type virus infection. In contrast, only HLA-C*01:02-YI9-4V tetramer-binding T cells were detected in all 4 GagV280 virus-infected individuals (Fig. 4C), suggesting that these T cells may have carried TCRs with a much stronger affinity for the HLA-C*01:02-YI9-4V complex than for HLA-C*01:02-YI9-4T. On the other hand, cross-reactive T cells having TCRs recognizing evenly both HLA-C*01:02-YI9-4T and HLA-C*01:02-YI9-4V were detected in patient VI-346 (Fig. 3B and E). Thus, cross-reactive T cells having a different TCR affinity for HLA-C*01:02-YI9-4T/4V may have been elicited in the consensus-type virus-infected individuals.

### Elicitation of YI9-specific T cells in HIV-1 subtype B virus-infected HLA-01:02^+^ Japanese individuals.

**FIG 5:**
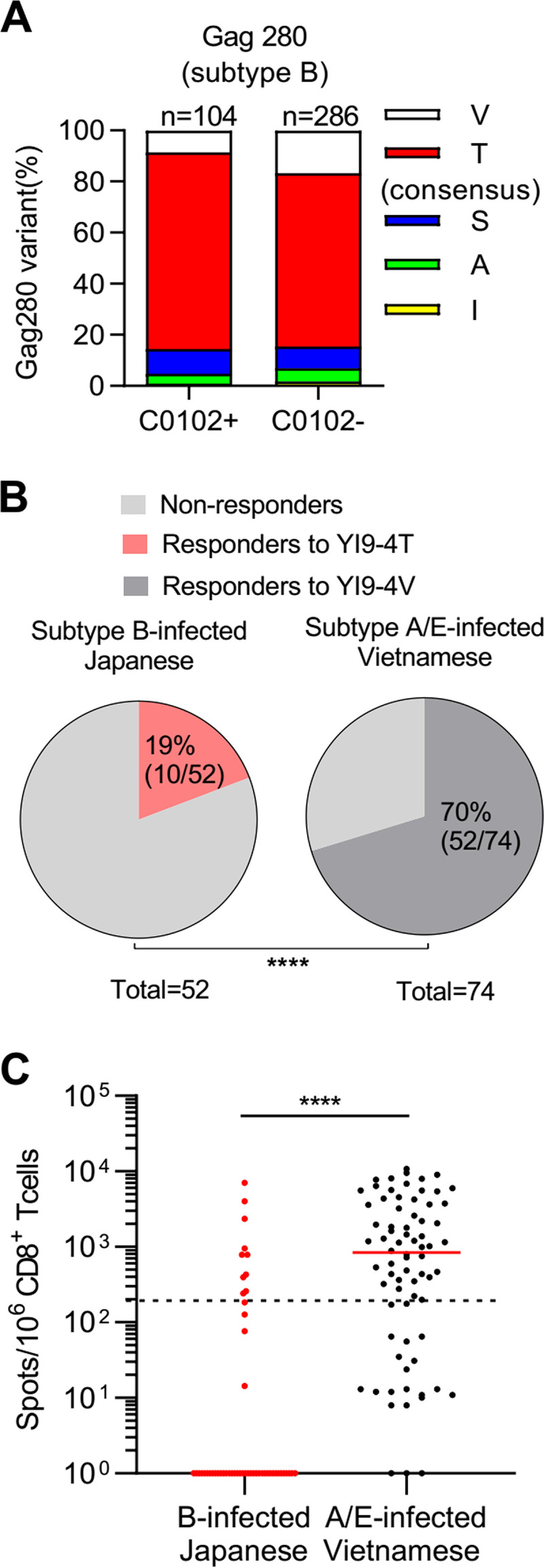
Comparison of T-cell responses to consensus-type YI9 epitope between HIV-1 subtype B-infected Japanese and subtype A/E-infected Vietnamese individuals. (A) Mutations at Gag280 in 390 HIV-1 subtype B-infected Japanese individuals. The amino acid sequence at Gag280 was compared between 104 HLA-C*01:02^+^ individuals and 286 HLA-C*01:02^–^ ones. V, S, A, I, and T were found at Gag280 in 9, 10, 4, 1, and 80 HLA-C*01:02^+^ individuals, respectively, whereas V, S, A, I, and T were detected at Gag280 in 48, 24, 15, 5, and 194 HLA-C*01:02^–^ individuals, respectively. (B and C) The T-cell responses to YI9-4T peptide in 52 subtype B-infected Japanese individuals were analyzed by performing an ELISPOT assay. The response was compared with that to YI9-4V in 74 subtype A/E-infected Vietnamese. The frequency of the responders and the magnitude of the response are shown in the pie charts (B and C, respectively). The dotted line at 200 spots/10^6^ CD8^+^ T cells indicates a threshold for a positive response (C). Statistical analysis was performed with the Fisher exact test (B) and Mann-Whitney tests (C), and the results are indicated. ****, *P* < 0.0001.

The frequency of HLA-C*01:02 in HIV-1 subtype B-infected Japanese individuals is approximately 27%, which is similar to that in the subtype A/E-infected Vietnamese individuals (13, 14). However, HLA-C*01:02-associated mutation at Gag280 was not found in Japanese individuals infected with the HIV-1 subtype B virus (13). We therefore reanalyzed the association of HLA-C*01:02 with the Gag280 sequence from 390 Japanese individuals chronically infected with the subtype B virus. The result showed no HLA-C*01:02-associated Gag280 mutations (Fig. 5A). We next investigated the frequency of HLA-C*01:02^+^ individuals who had YI9-4T consensus-type-specific T cells among the subtype B-infected HLA-C*01:02^+^ Japanese individuals and compared it to that of YI9-4V consensus-type-specific T cells in the subtype A/E-infected HLA-C*01:02^+^ Vietnamese. We analyzed the T-cell response to the YI9-4T peptide in 52 HLA-C*01:02^+^ Japanese individuals infected with the subtype B virus by performing the ELISPOT assay and compared the results to those for the response to the YI9-4V peptide in 74 HLA-C*01:02^+^ Vietnamese individuals infected with the subtype A/E virus. Nineteen percent of the Japanese were responders, whereas 70% of the Vietnamese individuals were responders (Fig. 5B). The magnitude of these T cell responses in the latter individuals was also much higher than that in the former ones (Fig. 5C). These findings suggest that YI9 consensus-type epitope YI9-4T was less immunogenic in the subtype B infection compared to the YI9 consensus-type epitope YI9-4V in the subtype A/E infection.

### Elicitation of RI8-specific T cells in HIV-1 subtype A/E-infected HLA-B*52:01^+^ Vietnamese individuals.

**FIG 6:**
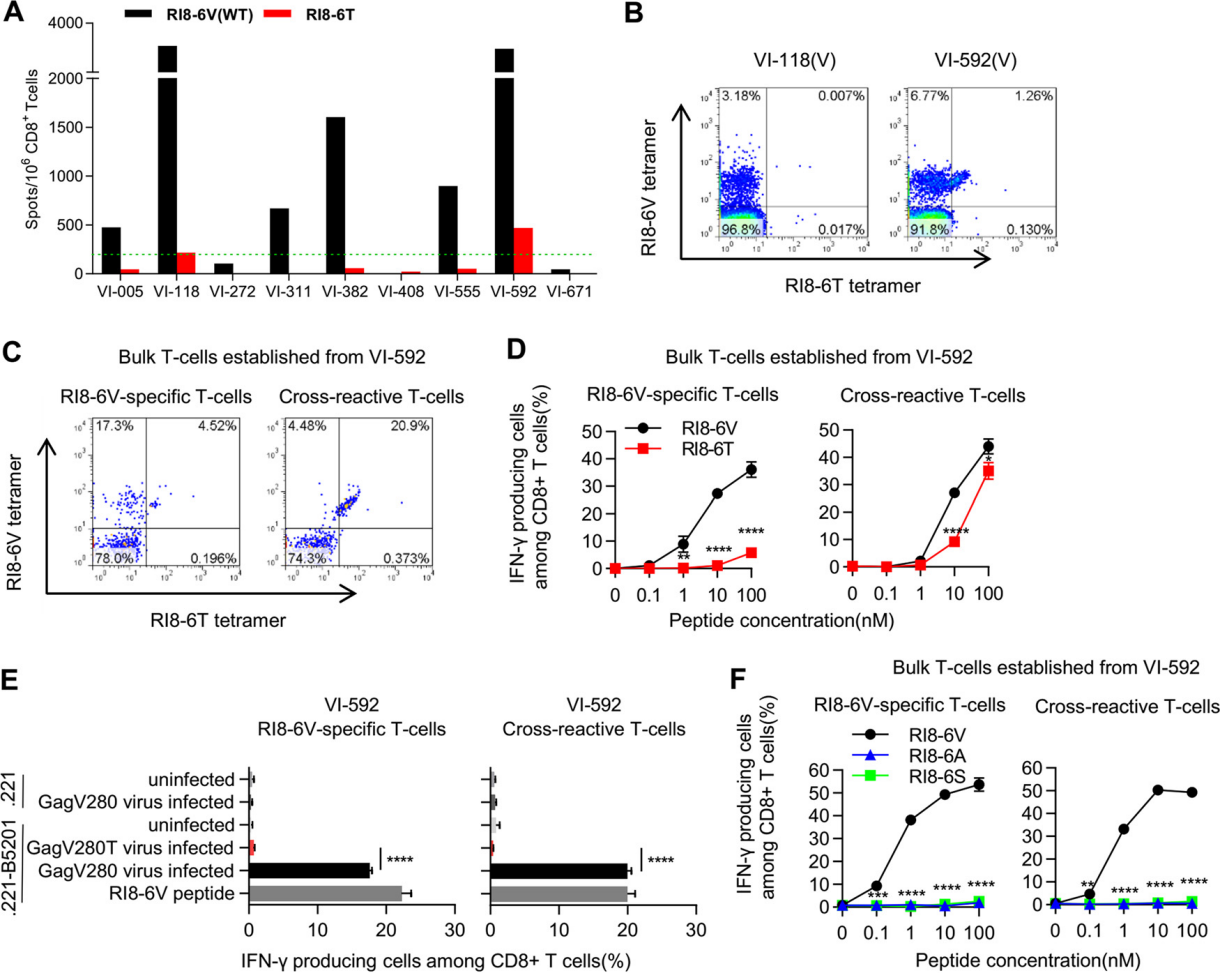
Elicitation of RI8-6V/6T-specific T cells in HLA-B*52:01^+^ Vietnamese individuals infected with the subtype A/E virus. (A) T-cell responses to RI8-6V and RI8-6T mutant peptides in nine HLA-B*52:01^+^ Vietnamese individuals infected with the subtype A/E virus. (B) Identification of RI8-specific T cells among PBMCs from VI-118 and VI-592. PBMCs were stained with both HLA-B*52:01-RI8-6V and HLA-B*52:01-RI8-6T tetramers. (C) Identification of RI8-specific T cells among CD8^+^ bulk T cells induced from PBMCs from patient VI-592 by stimulating the cells with RI8-6V or -6T peptide. Cells were stained with both HLA-B*52:01-RI8-6V and HLA-B*52:01-RI8-6T tetramers. (D and E) Recognition of RI8-6T mutant epitope by RI8-6V-specific T cells and cross-reactive ones. T-cell responses to 721.221-B*52:01 cells prepulsed with RI8-6V or -6T peptide (D) and those to those infected with 93JP-NH1-GagV280 virus or 93JP-NH1-GagV280T virus (E) were analyzed. The frequencies of p24-positive cells among 721.221-B*52:01 cells infected with 93JP-NH1-GagV280, those infected with 93JP-NH1-GagV280T, and those infected with 93JP-NH1-GagV280 were 40.3, 38.2, and 37.1%, respectively (D). (F) Recognition of RI8-6A and RI8-6S mutant epitopes by RI8-6V-specific T cells and cross-reactive ones. T-cell responses to 721.221-B*52:01 cells prepulsed with RI8-6V, RI8-6A, or RI8-6S peptide were analyzed. The results are given as means with the SD (*n* = 3). Statistical analysis was performed by using an unpaired *t* test. **, *P* < 0.01; ***, *P* < 0.001; ****, *P* < 0.0001.

From the finding that HLA-B*52:01-associated mutations were not detected at Gag280 in the subtype A/E virus-infected Vietnamese individuals, we speculated two possibilities: (i) weaker T cell responses to the RI8 epitope were elicited in the subtype A/E virus-infected Vietnamese individuals or (ii) HLA-B*52:01-associated mutations did not accumulate due to a lower frequency of HLA-B*52:01 in Vietnam. Indeed, the frequency of HLA-B*52:01 in Vietnam is only 3.7%. We investigated the elicitation of RI8-specific T cells in nine subtype A/E-infected HLA-B*52:01^+^ Vietnamese individuals by performing the ELISPOT assay to analyze their T cell responses to RI8-6V and RI8-6T peptides. Six of these individuals were responders to the RI8-6V consensus-type peptide, but two of these responders showed a weak response to the RI8-6T mutant peptide (Fig. 6A). Eight of these individuals were infected with the consensus-type subtype A/E virus, but the sequence data at Gag280 in patient VI-311 could not be obtained due to a very low plasma viral load (pVL). We further analyzed PBMCs from two individuals, VI-118 and VI-592, by staining them with HLA-B*52:01-RI8-6T and HLA-B*52:01-RI8-6V tetramers. We found a high frequency of RI8-6V-specific T cells in both individuals, whereas VI-592 also had cross-reactive T cells which bind to both tetramers (Fig. 6B). We next established bulk T cells from VI-592 by stimulating PBMCs from this patient with RI8-6V peptide or RI8-6T one and then culturing them for 2 weeks. Both RI8-6V-specific and cross-reactive bulk T cells were established by stimulation with RI8-6V peptide, whereas only cross-reactive bulk T cells were obtained by that with the RI8-6T peptide (Fig. 6C). The former T cells strongly recognized the RI8-6V peptide but not the RI8-6T peptide, whereas the latter recognized RI8-6V more so than RI8-6T (Fig. 6D). However, both T cells recognized GagV280 virus-infected cells but not GagV280T mutant virus-infected cells (Fig. 6E). These results support the idea that RI8-6V-specific T cells or the cross-reactive ones could select the GagV280T mutant subtype A/E virus. Therefore, the low frequency of HLA-B*52:01 in Vietnam may explain the fact that HLA-B*52:01-associated mutations had not accumulated in the A/E virus-infected Vietnamese. We further analyzed recognition of these T cells for RI8-6A and RI8-6S peptides. Both types of T cells failed to recognize these peptides (Fig. 6F).

### Viral-replication capacity of HIV-1 subtype A/E and B viruses having T/V mutation at Gag280.

**FIG 7:**
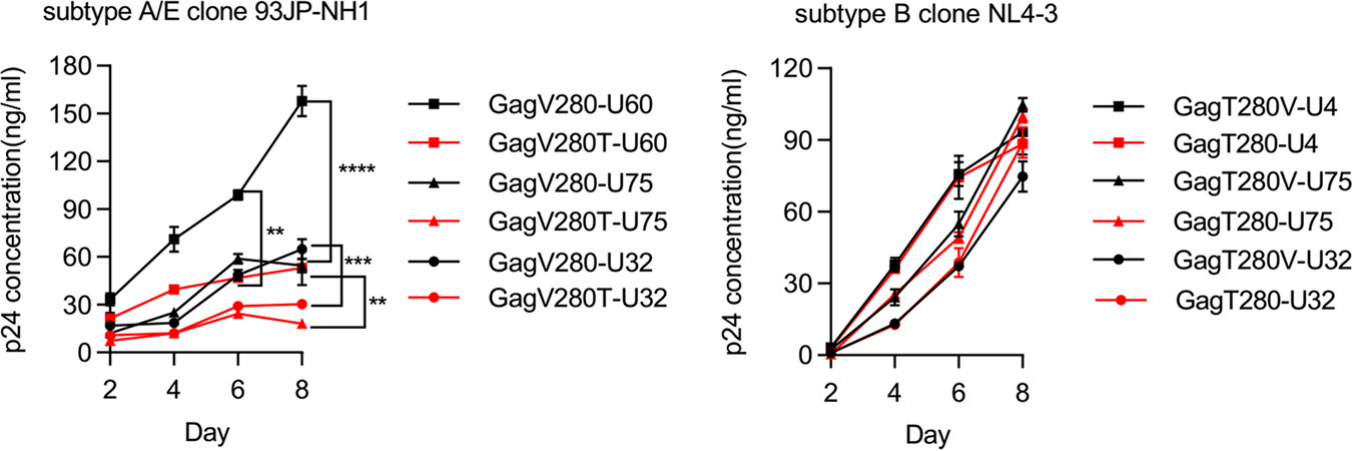
Replication kinetics of Gag280 mutant viruses in CD4^+^ T cells. *In vitro* viral replication analysis of Gag280 mutant virus was performed. Activated CD4^+^ T cells from three HLA-C*01:02^+^ donors (U-60, U-75, and U-32) were infected with 93JP-NH1-GagV280 or 93JP-NH1-GagV280T (left), while those from three HLA-B*52:01^+^ donors (U-4, U-75, and U-32) were infected with NL4-3-GagV280 or NL4-3-GagV280T (right). The concentration of p24 antigen in the culture supernatant was determined by using an enzyme immunoassay. All data are shown as means and SD (*n* = 3). Statistical analysis was performed by using the unpaired *t* test. **, *P* < 0.01; ***, *P* < 0.001; ****, *P* < 0.0001.

Next, we investigated the effect of the GagV280T mutation on the viral-replication capacity of the subtype A/E virus. The subtype A/E clone virus having the GagV280T mutation was generated by site-direct mutagenesis by using the subtype A/E clone 93JP-NH1 having Val at Gag280. CD4^+^ T cells isolated from 3 HLA-C*01:02^+^ healthy donors were used as target cells for 93JP-NH1 and its GagV280T mutant virus in the viral-replication capacity assay. The result showed that the subtype A/E virus with the GagV280T mutation had significantly reduced viral-replication capacity compared to the consensus-type one (Fig. 7A). We also analyzed the viral-replication capacity of the subtype B clone NL4-3 (Thr at Gag280) and its mutant virus NL43-GagT280V. The result showed that this capacity of both subtype B viruses was almost identical (Fig. 7B). These results together indicate that the effect of the Gag280 mutation on viral replication capacity different between the subtype A/E and subtype B viruses.

The low viral-replication capacity of the mutant subtype A/E virus suggests a reversion of the mutant A/E virus to the consensus-type one in HLA-C*01:02-negative individuals. Taken together with the selection of GagV280T mutant viruses by YI9-4V-specific T cells in HLA-C*01:02^+^ individuals, this also suggests a mechanism for the accumulation of HLA-C*01:02-associated GagV280T mutation in subtype A/E-infected individuals.

### Contribution of YI9-4V-specific or YI9-4T-specific CD8^+^ T cells to clinical outcome in the subtype A/E infection.

**FIG 8:**
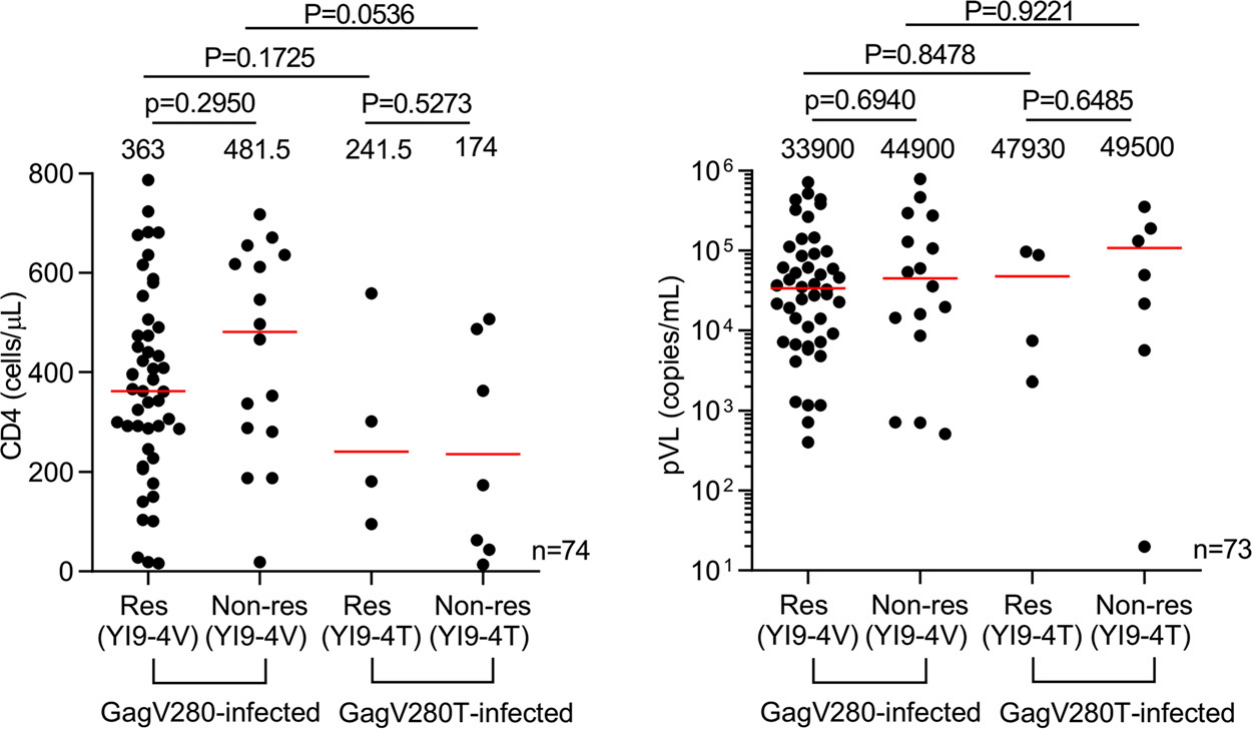
Comparison of clinical outcome between T-cell responders to YI9 and nonresponders. Comparison of CD4 count and pVL between the responders to the YI9-4V peptide epitope among the consensus-type virus-infected individuals and those to YI9-4T in GagV280T mutant virus-infected individuals and nonresponders among 74 subtype A/E-infected HLA-C*01:02^+^B*52:01^–^ Vietnamese. Statistical analysis was performed by using the Mann-Whitney test. The red lines in each figure represent the medians for CD4 count and pVL.

YI9-4V-specific T cells had strong ability to suppress replication of the consensus-type A/E virus *in vitro*, whereas YI9-4T-specific T cells had weak ability to suppress that of the mutant virus (Fig. 3F), suggesting that YI9-4V-specific T cells effectively suppress replication of this consensus-type A/E virus. We therefore analyzed the role of YI9-specific T cells in the clinical outcome of subtype A/E-infected HLA-C*01:02^+^B*52:01^–^ Vietnamese individuals. We found no significant difference in CD4 count or pVL between the responders to YI9-4V in the consensus-type virus-infected HLA-C*01:02^+^ individuals or in those to YI9-4T in GagV280T mutant virus-infected ones and nonresponders in the subtype A/E infection (Fig. 8). These findings cause a hypothesis that the YI9-4V-specific T cells did not have stronger ability to suppress HIV-1 replication *in vivo* compared to other T cells in these individuals. However, the mechanism explaining the discrepancy between *in vitro* and *in vivo* function of the T cells remains unknown.

## DISCUSSION

**FIG 9:**
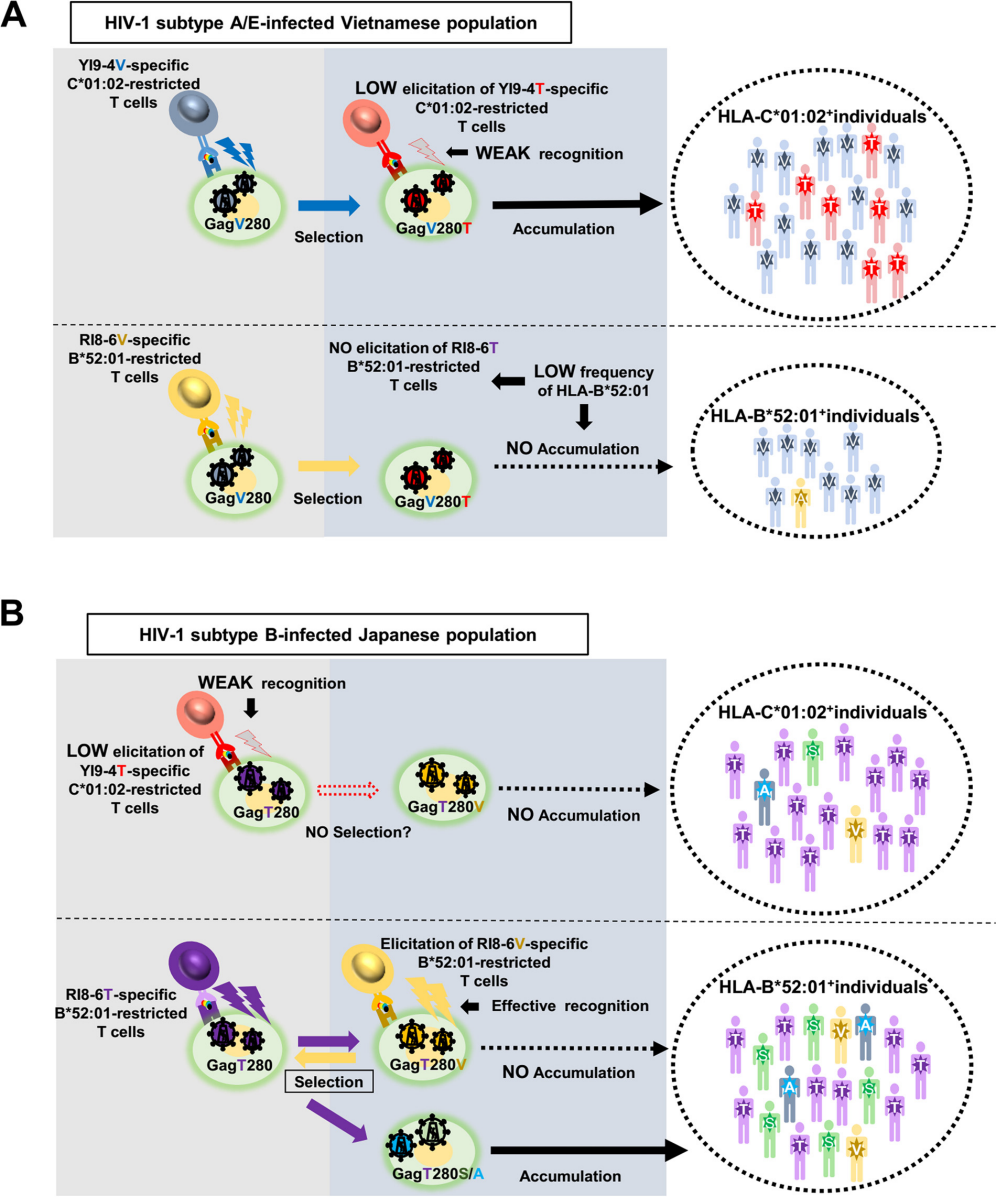
Summary of this study. (A) Accumulation and selection of Gag280 mutations in HIV-1 subtype A/E-infected Vietnamese individuals. (B) Accumulation and selection of Gag280 mutations in HIV-1 subtype B-infected Japanese individuals.

Previous studies demonstrated that HLA-C*01:02-associated Gag280 mutations are found in subtype A/E-infected Vietnamese individuals (14) but not in subtype B-infected Japanese ones (13). Since the frequencies of HLA-C*01:02 are 28.7 and 27.2% in the Vietnamese and Japanese individuals, respectively, this difference would not affect the difference in accumulation of HLA-C*01:02-associated Gag280 mutations between these two populations. On the other hand, we demonstrated that the difference in the consensus sequence at Gag280 between the subtype B and A/E viruses did affect the elicitation of HLA-C*01:02-restricted YI9-4V/4T-specific T cells and the recognition by these T cells of Gag280 mutations. YI9-4V-specific T cells were elicited in approximately 70% of the subtype A/E-infected HLA-C*01:02^+^ Vietnamese individuals. These T cells could select the Gag V280T mutant in the subtype A/E infection since they had a strong ability to suppress the replication of the consensus-type subtype A/E virus but failed to suppress that of the GagV280T mutant virus (Fig. 9A). In the subtype B virus-infected Japanese individuals, only 10% of them were infected with the GagT280V mutant virus. Since the frequency of HLA-C*01:02 is approximately 27% in Japanese individuals, it would be expected that effective YI9-4V-specific T cells would be elicited in only 2 to 3% of the subtype B-infected individuals and that the remaining 25% of the individuals, who were infected with the GagT280 consensus-type virus, would weakly respond to the YI9-4T epitope. Indeed, only 19% of HLA-C*01:02^+^ Japanese individuals infected with the subtype B virus were responders to YI9-4T. These findings suggest that HLA-C*01:02-restricted YI9-4T-specific T cells were not involved in the accumulation of Val at Gag280 in the Japanese individuals infected with the subtype B virus (Fig. 9B).

A cross-reactive T cell line from patient VI-346 evenly recognized both YI9-4T and YI9-4V peptides, and the binding affinity of the 2 C*01:02-tetramers to the T cell line was identical. These findings strongly suggest that the cross-reactive T cell line carried TCRs with the same affinity for both HLA-C*01:02-YI9-4V/4T molecules. This T cell line recognized target cells infected with the GagV280 consensus-type virus more effectively than it did those infected with the GagV280T virus, whereas it suppressed the replication of the former virus but not that of the latter one. These results together indicate that the antigen presentation of the YI9-4T peptide in GagV280T mutant virus-infected cells was reduced compared to that of the YI9-4V peptide in GagV280 consensus-type virus-infected cells. Reduced antigen presentation of YI9-4T peptides may have resulted in a weak elicitation of HLA-C*01:02-restricted YI9-4T-specific T cells in HLA-C*01:02^+^ individuals infected with the subtype A/E GagV280T virus or with the consensus-type subtype B virus. YI9-4T-specific T cells recognized GagV280T mutant virus-infected cells more than did the cross-reactive T cells, whereas the ability of these YI9-4T-specific T cells to suppress the replication of GagV280T mutant viruses was weaker than that of YI9-4V-specific T cells to suppress the replication of the consensus-type ones (Fig. 3C and F). The weak recognition of the mutant-specific T cells for GagV280T virus-infected cells may have led to the accumulation of GagV280T mutant virus in the HLA-C*01:02^+^ Vietnamese individuals.

HLA-B*52:01-associated Gag280 mutations have not been analyzed in subtype A/E-infected Vietnamese individuals due to a low frequency of HLA-B*52:01 (3.7% in the subtype A/E-infected Vietnamese). The present study demonstrated that HLA-B*52:01-restricted RI8-6V-specific T cells were effectively elicited in the subtype A/E-infected HLA-B*52:01^+^ individuals and recognized cells infected with the consensus-type virus. These findings suggest that RI8-6V-specific T cells had the ability to select GagV280T mutant subtype A/E viruses. Therefore, failed accumulation of HLA-B*52:01-associated Gag280 mutations in HIV-1-infected Vietnamese might be explained by the low frequency of HLA-B*52:01 in them. Thus, the role of the HLA-B*52:01-restricted T cells on the accumulation of GagV280T would be minimal in the subtype A/E-infected Vietnamese individuals. These findings together suggest that GagV280T was selected by the HLA-C*01:02-restricted T cells rather than by the HLA-B*52:01-restricted T cells in the subtype A/E-infected Vietnamese (Fig. 9A).

The analysis of viral-replication capacity by using subtype A/E clone 93JP-NH1 showed that the subtype A/E virus with the GagV280T mutation had reduced viral replication capacity compared to that for the consensus-type virus. The consensus sequence of Gag280 in the HIV-1 subtype C virus was Val. A recent study demonstrated a reduced viral replication capacity of the subtype C virus having a GagV280T/S/A mutation compared to that of the consensus-type subtype C virus (33). This present study also showed no significant difference in viral-replication capacity between the subtype B virus (NL4-3) having GagT280 and the mutant virus having GagT280V. Thus, the effect of Gag280 mutations on viral-replication capacity was different among HIV-1 subtypes. The reduced viral-replication capacity in subtype A/E and C viruses may have contributed to the accumulation of these mutations in HLA-C*01:02^+^ and HLA-B*52:01^+^ individuals infected with subtype A/E and C viruses, respectively.

The expression level of HLA-C molecules on cells is lower than that of HLA-A or HLA-B ones (34, 35). The lower expression of HLA-C is found even on HIV-1-infected cells in which HLA-A and HLA-B molecules are downregulated by Nef (36). These findings suggest that HLA-C-restricted T cells may be less sufficiently elicited in HIV-1-infected individuals. A previous study showed that the expression level of HLA-C alleles is positively correlated with the frequency of HLA-C-restricted HIV-1-specific T-cell responses (37). YI9-4V-specific T cells were detected in 74.6% of HLA-C*01:02^+^ Vietnamese individuals infected with the consensus-type virus. Since HLA-C*01:02 is expressed on the cell surface as the second highest among 16 HLA-C alleles in African-Americans (37), higher expression of HLA-C*01:02 may account for effective elicitation of YI9-4V-specific T cells in these individuals.

In the present study, we demonstrated that the GagYI9-4V-specific T cells, which were frequently elicited in the individuals infected with the consensus-type subtype A/E virus, failed to recognize GagV280T mutant A/E virus-infected cells, whereas GagYI9-4T mutant epitope-specific T cells, which were weakly elicited in individuals infected with GagV280T mutant A/E virus, had weak or no ability to recognize the mutant virus. These results suggest a mechanism for selection and accumulation of GagV280T mutants in a subtype A/E infection (Fig. 9A). A previous study showed that HLA-B*52:01-restricted RI8-6T-specific T cells can select GagV280A/S mutations in subtype B-infected Japanese individuals (13, 30). HLA-B*52:01-restricted RI8-6V-specific T cells are elicited in subtype A/E-infected HLA-B*52:01^+^ Vietnamese individuals and these T cells recognized target cells infected with the consensus-type virus but failed to recognize RI8-6A/S mutant epitopes (Fig. 6E and F). These findings imply that the RI8-6V-specific T cells can select the GagV280A/S mutations. Therefore, it is likely that GagV280A/S mutations did not accumulate in the population due to the low frequency of HLA-B*52:01 in Vietnam. On the other hand, HLA-C*01:02-restricted GagYI9-4T-specific T cells were weakly elicited in individuals infected with the subtype B virus having the GagT280 consensus sequence, leading to no selection of the GagT280V mutant virus. HLA-B*52:01-restricted RI8-6T/6V-specific T cells were effectively elicited in both subtype B-infected and subtype A/E-infected individuals. Thus, the difference in consensus sequence affected the elicitation of HLA-C*01:02-restricted GagYI9-specific T cells but not that of HLA-B*52:01-restricted RI8-specific T cells. These findings account for the difference in the accumulation of HLA-associated Gag280 mutations between the subtype A/E and B infections.

A previous study on HLA-APs in Uganda revealed that 34% of the identified HLA-associated polymorphisms were significantly and differentially selected between subtypes A1 and D (16). This study showed only that the subtype A1 consensus peptide had stronger affinity for HLA-B*15:03 than the subtype D consensus peptide, as found from the analysis of one case of these HLA-APs, Nef K105R, which is selected due to the presence of putative HLA-B*15:03-restricted epitope NefRL9 in subtype A1 but not subtype D. Thus, this study implied that the difference in consensus sequence affects the selection of CTL escape mutations. In the present study, we demonstrated that the difference in consensus sequence between the subtype B and A/E viruses influenced the elicitation of the GagYI9-specific T cells and the recognition of the mutant virus, leading to the difference in the accumulation of HLA-associated Gag280 mutations. The result suggests that an HIV-1 vaccine using antigens having different consensus sequences may influence elicitation of effective T cells for protection against an HIV-1 infection. In this respect, analysis of the immunogenicity of HIV-1 vaccines using chimeric antigens in conserved regions covering different HIV-1 subtypes is important to evaluate the vaccines (38–41). The present study also has an impact on the development of HIV-1 vaccines covering different HIV-1 subtype viruses.

## MATERIALS AND METHODS

### Subjects.

Treatment-naive Vietnamese individuals chronically infected with subtype A/E were recruited from the National Hospital of Tropical Diseases, Vietnam. This study was approved by the Ethics Committee of the Vietnamese Ministry of Health (no. 2342/OD-BYT). Treatment-naive Japanese individuals chronically infected with HIV-1 subtype B were recruited from the National Center for Global Health and Medicine, Japan. This study was approved by the ethics committees of Kumamoto University (RINRI-1340 and GENOME-342) and the National Center for Global Health and Medicine (NCGM-A-000172-01). Three HLA-C*01:02^+^ healthy donors were recruited for this study, which was approved by the Ethical Committee of Kumamoto University, Japan. Informed consent was obtained from all individuals according to the Declaration of Helsinki. PBMCs were separated from whole blood. HLA types of HIV-infected individuals were determined by standard sequence-based genotyping. The pVLs of the individuals at their first visit were measured by using the Cobas TaqMan HIV-1 real-time PCR version 2.0 assay (Roche Diagnostics, NJ).

### Cell lines.

721.221 cells expressing CD4 molecules and HLA-C*01:02 (721.221-C*01:02) or HLA-B*52:01 (721.221-B*52:01) were previously generated (42, 43). RMA-S cells expressing HLA-C*01:02 were generated by the introduction of HLA-C*01:02 genes into RMA-S cell lines. These cells were maintained in RPMI 1640 medium (Invitrogen) containing 5% fetal calf serum (R5) and 0.15 mg/ml of hygromycin B or 0.2 mg/ml neomycin. The NP2/CD4 cell line expressing CXCR4 with the long terminal repeat (LTR)-driven luciferase gene (44) was provided by Y. Maeda, Department of Microbiology, Kumamoto University, Kumamoto, Japan.

### HIV-1 mutant clones.

NL4-3 mutant (NL4-3-GagT280V) was previously generated (30). 93JP-NH1 mutant (93JP-NH1-GagV280T) was generated by introducing the GagV280T mutation into 93JP-NH1 by use of a previously described site-directed mutagenesis system (45).

### Identification of HIV-1 subtype and YI9 epitope sequence.

To identify HIV-1 subtype (subtype B and subtype A/E), the sequences of whole Gag from HIV-1-infected individuals were analyzed by using the Recombinant Identification Program (RIP 3.0; https://www.hiv.lanl.gov/content/sequence/RIP/RIP.html). Determination of the epitope sequence for YI9 was performed as previously described (13, 14). The YI9 sequence data from 386 chronically HIV-1 subtype A/E-infected treatment-naive Vietnamese individuals were analyzed after excluding 4 individuals having a mixture amino acid sequence at Gag280 from previous analyzed ones and adding 21 new individuals (14). Amino acid sequence data at Gag280 were collected from Gag sequence data previously identified from 390 Japanese individuals infected with the subtype B virus (13, 31).

### IFN-γ ELISPOT assay.

*Ex vivo* gamma interferon (IFN-γ) ELISPOT assays were performed as previously described (30, 40). The number of spots for a T-cell response to each peptide was finally calculated by subtracting the number of spots in wells without peptides from that of spots with peptides. The mean (plus 3 standard deviations [SD]) spot number of samples from 13 HIV-1-naive individuals for the peptides was 162 spots/10^6^ CD8^+^ T cells (30, 40). Therefore, we defined >200 spots/10^6^ CD8^+^ T cells as positive responses.

### Tetramer staining.

HLA-C*01:02-YI9-4V/4T tetrameric complexes (tetramers) were generated as previously described (46, 47). HLA-B*52:01-RI8-6T/6V tetrameric complexes (tetramers) were previously generated (31). PBMCs or HIV-1-specific T-cell lines were stained at 37°C for 30 min with a combination of APC-conjugated YI9-4V and phycoerythrin (PE)-conjugated YI9-4T-HLA-C*01:02 tetramers or a combination of PE-conjugated RI8-6T and allophycocyanin (APC)-conjugated RI8-6V-HLA-B*52:01 tetramers at 100 nM. For determination of the binding affinity of TCRs, the cross-reactive T cells were stained with YI9-4V and YI9-4T-HLA-C*01:02 tetramers at various concentrations at 37°C for 30 min. The cells were subsequently stained with fluorescein isothiocyanate (FITC)-conjugated anti-CD3 (Dako, Glostrup, Denmark), Pacific Blue-conjugated anti-CD8 monoclonal antibody (MAb; BD Biosciences), and 7-AAD (BD Pharmingen) at 4°C for 30 min and analyzed with a FACS-Canto II (BD Bioscience, CA). The frequency of HLA-tetramer^+^ cells was measured after gating the CD3^+^ CD8^+^ population.

### Generation of epitope-specific T-cell lines.

PBMCs were stained with PE- or APC-conjugated tetramers, FITC-conjugated anti-CD3 (Dako), Pacific Blue-conjugated anti-CD8 MAb (BD Biosciences), and 7-AAD (BD Pharmingen), after which CD3^+^ CD8^+^ 7-AAD^–^ tetramer^+^ T cells were sorted in U-bottomed 96-well microtiter plates (100 to 500 cells/well for T-cell lines) by using a FACS Aria (BD Biosciences). The sorted cells were stimulated with corresponding epitope peptide and cultured as previously described (47). For the RI8-specific bulk T cells, PBMCs were stimulated with specific RI8-6V or -6T peptide (100 nM). After 2 to 3 weeks in culture, epitope-specific CD8^+^ T cells were used in functional assays after their purity had been confirmed by flow cytometry analysis using tetramers.

### Intracellular cytokine staining assay.

721.221 cells prepulsed with HIV-1 epitope peptide or 721.221 cells infected with HIV-1 virus were cocultured with T-cell clones or lines in a 96-well plate for 2 h at 37°C. Brefeldin A (10 μg/ml) was then added, and the cells were incubated further for 4 h at 37°C. The cells were then fixed with 4% paraformaldehyde and incubated in permeabilization buffer (0.1% saponin, 10% FBS, phosphate-buffered saline) and then were stained with APC-conjugated anti-CD8 MAb (Dako), followed by FITC-conjugated anti-IFN-γ MAb (BD Biosciences). The percentage of IFN-γ-producing cells among the CD8^+^ T-cell population was determined by using the FACS-Canto II.

### HLA stabilization assay.

The affinity of peptide binding to HLA-C*01:02 was examined by using RMA-S-C*01:02 cells, as previously described (48, 49). Briefly, these RMA-S transfectant cells were cultured at 26°C for 16 h, then pulsed with peptides at 26°C for 1 h, and subsequently incubated at 37°C for 3 h. Staining of the cell surface HLA molecules was performed by using HLA-C-specific MAb DT-9 (36) and FITC-conjugated sheep anti-mouse IgG (Jackson ImmunoResearch). The fluorescence intensity was measured with the FACS-Canto II.

### HIV-1 replication suppression assay.

The ability of epitope-specific CD8^+^ T cells to suppress HIV-1 replication was measured as described previously (45, 50). CD4^+^ T cells isolated from HLA-matched healthy donor PBMCs were infected with HIV-1 virus and then cocultured with epitope-specific T cells at E:T ratios of 1:1, 0.1:1, and 0:1. On day 5 postinfection, the concentration of p24 antigen in the culture supernatant was measured by using an enzyme-linked immunosorbent assay kit (HIV-1 p24 Ag ELISA kit; ZeptoMetrix). The percentage of suppression was calculated as follows: [(concentration of p24 without CTLs – concentration of p24 with CTLs)/concentration of p24 without CTLs] × 100.

### Replication kinetics assay.

The replication kinetics of the NL43-GagT280 and NL43-GagT280V viruses were examined as previously described (51, 52). CD4^+^ T cells isolated from HLA-matched healthy donor PBMCs were cultured for 5 to 7 days in wells coated with anti-human CD3 MAb (clone OKT3). After activated CD4^+^ T cells (2 × 10^5^) had been exposed to NL43-GagT280 or NL43-GagT280V infectious virus (500 blue cell-forming units in MAGIC-5 cells), the cells were cultured in 200 μl of R10 containing 1% nonessential amino acid solution and 1% 100 mM sodium pyruvate (complete medium) plus 200 U/ml of rIL-2. For the subtype A/E clone viruses, 93JP-NH1-GagV280 and -GagV280T, the CD4^+^ T cells (2 × 10^5^/well) were infected with 6 × 10^6^ relative luminescence units (RLU) of either 93JP-NH1-GagV280T or -GagV280 virus. The culture supernatant was collected from day 2 to day 8 postinfection, and the concentration of supernatant p24 antigen was measured by ELISA. The appropriate RLU were determined by infecting a NP2/CD4 cell line expressing CXCR4 with the LTR-driven luciferase gene (44) at various titers of virus, and then the RLU were measured by using a luciferase assay system (Promega).

### Statistical analysis.

The frequency of the mutation between HLA^+^ and HLA^–^ individuals was statistically analyzed by using a Fisher exact test. Groups were compared by performing the unpaired *t* test or two-tailed Mann-Whitney U tests. *P* values of <0.05 were considered significant.
